# Evaluation of the Geometric and Dosimetric Accuracy of Synthetic Computed Tomography Images for Magnetic Resonance Imaging-only Stereotactic Radiosurgery

**DOI:** 10.7759/cureus.4404

**Published:** 2019-04-08

**Authors:** Ali Fatemi, Madhava R Kanakamedala, Claus Chunli Yang, Bart Morris, William N Duggar, Srinivasan Vijayakumar

**Affiliations:** 1 Radiation Oncology, University of Mississippi Medical Center, Jackson, USA

**Keywords:** stereotactic radiosurgery, synthetic ct, convolution algorithm, magnetic resonance imaging (mri), computed tomography (ct), inhomogeneity correction

## Abstract

Introduction

Stereotactic radiosurgery (SRS) plans created using synthetic computed tomography (CT) images derived from magnetic resonance imaging (MRI) data may offer the advantage of inhomogeneity correction by convolution algorithms, as is done for CT-based plans. We sought to determine and validate the clinical significance and accuracy of synthetic CT images for inhomogeneity correction in MRI-only stereotactic radiosurgery plans for treatment of brain tumors.

Methods

In this retrospective study, data from two patients with brain metastases and one with meningioma who underwent imaging with multiple modalities and received frameless SRS treatment were analyzed. The SRS plans were generated using a convolution algorithm to account for brain inhomogeneity using CT and synthetic CT images and compared with the original clinical TMR10 plans created using MRI images.

Results

Synthetic CT-derived SRS plans are comparable with CT-based plans using convolution algorithm, and for some targets, based on location, they provided better coverage and a lower maximum dose.

Conclusions

The results suggest similar dose delivery results for CT and synthetic CT-based treatment plans. Synthetic CT plans offered a noticeable improvement in target dose coverage and a more gradual dose fall-off relative to TMR10 MRI-based plans. The major disadvantage is a slightly increased dose (by 0.37%) to nearby healthy tissue (brainstem) for synthetic CT-based plans relative to those created using clinical MRI images, which may be a problem for patients undergoing high-dose treatment.

## Introduction

Magnetic resonance imaging (MRI) is rapidly becoming one of the most important imaging modalities used in stereotactic radiosurgery (SRS) treatment planning for intracranial tumors. SRS is used to correct functional abnormalities and address small tumors of the brain. It can deliver precisely targeted radiation in fewer high-dose treatments than traditional therapy, an approach which can help preserve healthy tissue [1]. During the planning process, MRI and computed tomography (CT) images, and recently, cone beam computed tomography (CBCT) images, are acquired for delineation of tumor and organs at risk (OAR).

The convolution algorithm has recently been introduced as an optimized radiation planning tool in the Gamma Knife planning system. Its use allows correction for brain tissue inhomogeneity and provides more accurate dose calculation. CT images can be used with these algorithms; they provide electron density maps for convolution-based dose calculation and can be co-registered with MR images as a primary dataset to correct for geometric distortion [2]. The use of CT images alone for planning is not ideal due to their lack of quality soft tissue contrast; however, the process of registration with MRI images is associated with some significant uncertainties. CBCT reference images can be used for geometrical distortion correction and pre-treatment positioning on a Gamma Knife Icon machine (Leksell Gamma Knife Icon, Stockholm, Sweden) [2, 3].

Precise target definition is essential for successful SRS treatment. MRI provides superior soft-tissue contrast, physiological information, and multiplanar imaging capabilities, making it an ideal modality for target localization. Therefore, in general, the contours from MRI images are transferred to CT or CBCT images for treatment planning after fusion [4, 5]. However, multi-modality imaging is time-consuming for patients and can introduce systematic positioning errors of up to 2 mm during scanning and fusion, both reducing treatment accuracy and increasing patients’ exposure to imaging radiation [5].

The use of MRI images alone for radiation treatment planning (RTP), and especially SRS planning, has some limitations, including a lack of electron density information for accurate dose calculation, geometrical distortion of MRI images which affect the accuracy of target definition, and finally, patient localization as a result of lack of bony structure verification during patient setup on the machine [6]. Thus, CT is still the standard imaging modality of choice for treatment planning, and in SRS planning, if convolution is used for correction of tissue inhomogeneity rather than a tissue maximum ratio (TMR) algorithm [7-9].

Many groups are currently working on generating synthetic CT images derived from MRI images to address the first two limitations of RTP based on MRI images alone. This new process will potentially reduce workload, cost, geometrical mismatch associated with the co-registration process, and finally, the patient’s radiation exposure [7, 10-12].

There are different approaches to generate synthetic CT images. One of the simplest and most straightforward is a segmentation-based approach, which involves manual segmentation of each region-of-interest (ROI) over bony anatomy, soft tissue (fat and water), and air on MR images, with assignment of a bulk density to each region [13-15]. The accuracy of this technique has been evaluated for simple 3D conformal, intensity-modulated radiation therapy (IMRT) and SRS plans and has shown good dosimetric agreement for simple 3D conformal plans. However, dose deviations of up to 5% have been reported for IMRT head and neck cancer plans, as well as unacceptable values for SRS brain metastasis plans [16, 17].

The second approach uses an MRI atlas created from paired, co-registered MR-CT datasets to generate a conjugate electron density atlas. One common drawback in this technique is errors arising from mis-registration of atlas data with patient data; this issue is especially common in patients with surgical implants and/or other anatomical abnormalities, which are very common in post-operation radiotherapy patients [18, 19].

Finally, the third approach is the classification-based approach used in this study. This technique aims to classify or cluster different tissue types (i.e., muscle, adipose tissue, and bone) based on pixel intensities from multiple MR pulse sequences. One common problem with this technique is that in order to allow absolute comparison between two patients undergoing the same treatment, the same scanner and parameters (i.e. magnet strength, radiofrequency (RF) coil setup, and MRI pulse sequence) must be used because of the dependency of voxel signal intensity on these factors. Nonetheless, promising results using this technique have been reported for head, neck, and brain tumors [20, 21].

In current practice, during SRS planning using MRI images as a primary dataset, any tissue heterogeneity within the skull is ignored when using the TMR10 algorithm, even though the convolution algorithm is available for such correction. One of the drawbacks of using this algorithm is its speed and potential delay in patient treatment time. While the change in dose distribution when comparing the convolution and TMR10 algorithms is known to an extent, the specific changes that may lead to clinically significant differences are not well understood [22, 23].

Therefore, in this paper, we propose a method for MRI-only SRS planning using synthetic CT images using a classification-based approach, derived directly from MRI images. We investigated the accuracy and dosimetric differences between synthetic CT and CT-based plans created using a convolution-based algorithm, chosen to account for brain tissue inhomogeneity. The SRS treatment plans created in this way were compared with clinical MRI-based plans created using the TMR10 algorithm.

## Materials and methods

The study was approved by the University of Mississippi Medical Center institutional review board. In this retrospective study, we analyzed data from four patients (two with brain metastases and one with meningioma), who had undergone MRI, CT and CBCT scans and received frameless SRS treatment. As part of our clinical workflow for each patient we planned on MRI images co-registered with CT, and in some cases CBCT, for geometric distortion correction. Each plan may include multiple target brain metastases, and some patients had multiple plans with different isocenters. We used all plans in this analysis: eight plans from four patients, for a total of 11 brain metastasis, brainstem, and meningioma target tumors. The specific lesion sites varied from patient to patient.

Synthetic CT images were generated from MR images using syngo.via RT Image Suite (Siemens Healthineers, Erlangen, Germany) using a fuzzy c-means method. Note that the patient setup in MRI was different from that for CT and treatment. Experienced physicians rigidly registered the CT and MRI image datasets and defined the target volume on MRI images, and a medical physicist created SRS treatment plans using the TMR10 algorithm on MRI images. SRS doses were prescribed in the range of 13 to 20 Gray (Gy) to 50% to 75% isodose lines according to the lesion sizes. SRS plans were prepared using Leksell GammaPlan Elekta. In the next step, the volumes were transferred to synthetic CT and CT images. This information was used for planning on both synthetic CT and CT datasets using the convolution algorithm, with the same dose prescription as on primary MRI images. Dose maps were calculated on the synthetic CT and on the original CT, using the same plan parameters.

CT images were acquired using a Siemens SOMATOM Definition AS CT scanner (Siemens Healthineers, Erlangen, Germany) at 120 kVp and 220 mA with the patient in treatment position; each 0.6-mm-thick slice had a resolution of 512×512 pixels, with pixel spacing of 0.586 mm.

MR images were acquired using a Siemens Aera 1.5T diagnostic magnet (Siemens Healthineers, Erlangen, Germany). High-resolution magnitude and phase images were acquired for field mapping after automated shimming over the entire head volume, distortion correction filter on for system inherited gradient non-linearity correction and (Time of Echo 1 (TE1) /Time of Echo 2 (TE2)/Time of Repetition (TR) = 2.46/7.38/12 ms, 550 Hz/pixels, 1 mm^3^ isotropic sagittal 3D acquisition, standard 20-channel head coil).

**Figure 1:**
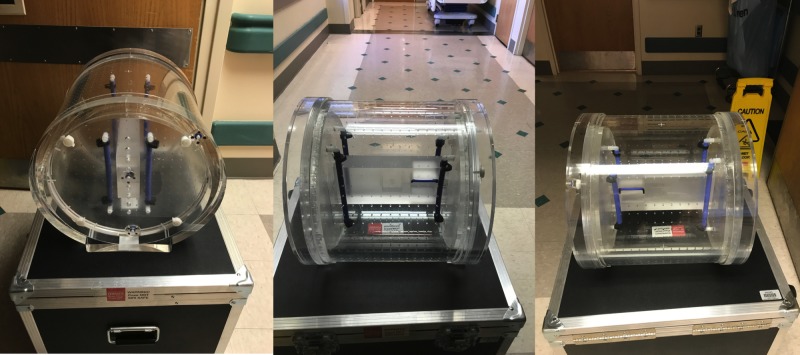
The QUASAR MRID3D phantom used to create machine-specific distortion maps

Phase images were subtracted and unwrapped to produce field maps (in-house software, IDL 8.2, Boulder, CO, USA and Mathworks, Natick, MA, USA). We derived the final displacement map to correct for residual geometrical distortion using a machine-related distortion map created using a QUASAR MRID^3D^ phantom (Modus Medical Devices Inc., London, Ontario, Canada) (Figure 1) and a B_0_-derived map. The final displacement map was applied to all MRI images after rescaling using the open-source Analysis of Functional Neuroimaging (AFNI) software package for brain imaging.

All patients were scanned, post-contrast, with a three-dimensional, T1-weighted, gradient-echo sequence (T1-MPRAGE), 1 mm^3^ isotropic axial 3D acquisition, bandwidth = 160 Hz/pixel, TR= 2200 ms, TE = 3.74 ms, T1 Vibe Dixon, voxel size: 1.0 x 1.3 x 1.3 mm (to generate images of fat and water), TR = 4.10 ms, TE1/TE2 = 1.23 ms/2.46 ms, Flip Angel (FA) = 9 degrees, bandwidth = 1090 Hz/pixel. T2 SPACE, voxel size 1 mm isotropic (anatomical scan appropriate for contouring), TR = 3200 ms, TE = 410 ms, bandwidth = 751 Hz/pixel PETRA, isotropic 1 mm, TR =3.32 ms, TE = 0.07 ms, FA = 6 degree, sagittal, bandwidth = 403 Hz/pixel (identification of air for the purpose of defining an air mask to exclude such voxels from calcification), voxel size 1 mm isotropic, vascular imaging (T2-weighted gradient echo, appropriate to create a threshold-based intensity mask to separate flowing blood), voxel size 3 mm, matrix: 256 x 230, TR = 8.6 ms, TE = 4 ms, FA = 20 degree, bandwidth = 320 Hz/pixel.

To study the accuracy and differences of synthetic CT plans created using the convolution algorithm, we initially visually checked the similarity of dose distribution calculated from both CT and synthetic CT images and compared it qualitatively with dose distribution for TMR10-based MRI plans. Then, by quantitively examining clinically relevant metrics, such as D100 (Gy), D95 (Gy), maximum, minimum, and mean dose were extracted for comparison of dose to target (brain metastasis or meningioma tumor) and OAR (brainstem).

## Results

**Figure 2:**
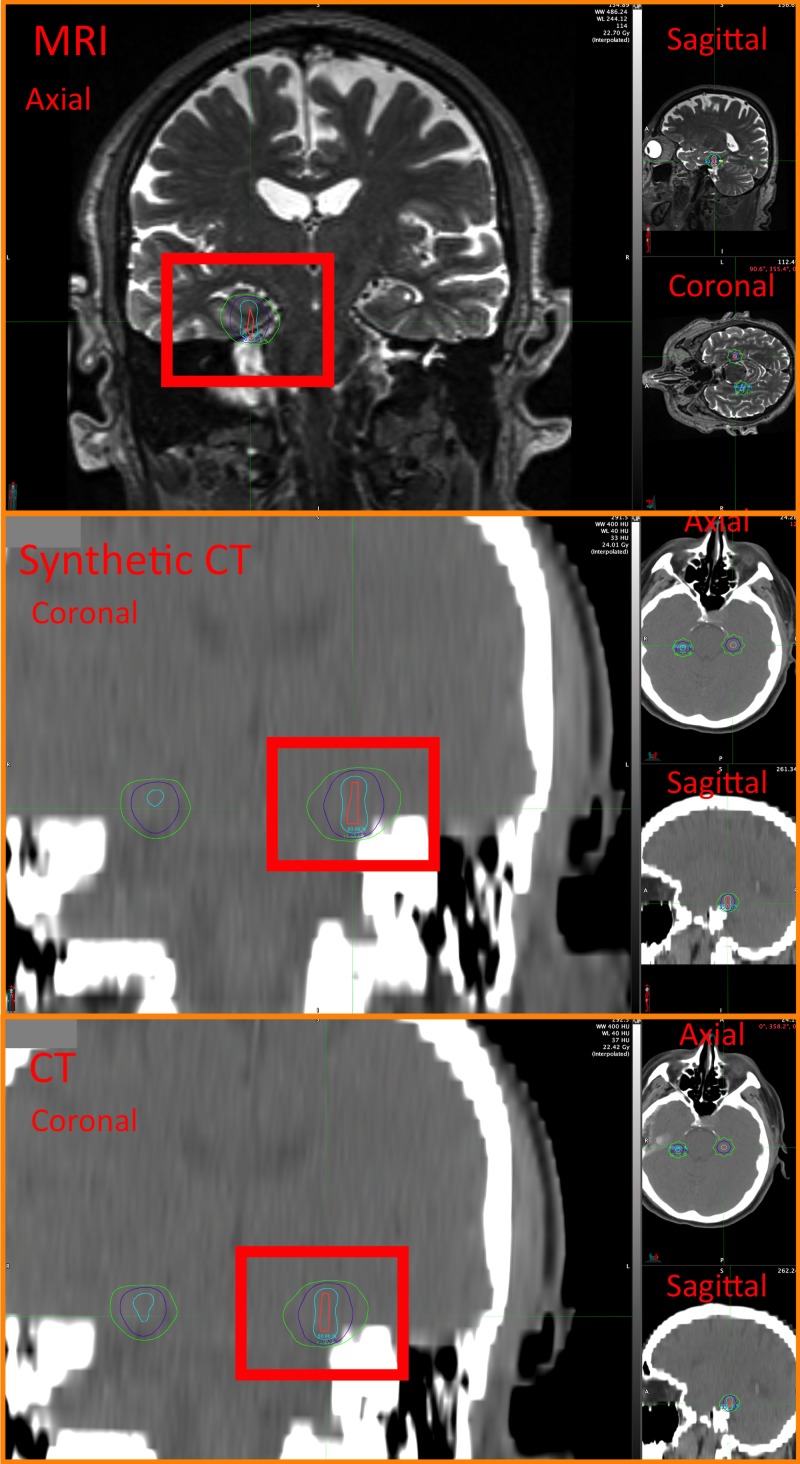
Representation of SRS plans for single metastasis brain tumor (orange color) at multiple planes (axial, sagittal, and coronal) in MRI images (top) using TMR (10) algorithm and synthetic CT (middle) and CT (bottom) using convolution algorithm. The 50% isodose line (cyan), 20% isodose line (purple) and 12% isodose line (green) has been demonstrated for all images and plans. Gy = Gray; TMR = Tissue Max Ratio; CT = Computed Tomography; MRI = Magnetic Resonance Imaging ; SRS = Stereotactic Radiosurgury

We demonstrate the 2D dose distribution for one of the brain metastases on three planes as an example (Figure 2). Dose distributions appeared similar for both CT and synthetic CT plans and were more conformal for both compared with TMR10-derived plans. Overall, the quantitative data (Table 1) show a 1.84% decrease in maximum point dose inside the targets for synthetic CT and a 1.77% decrease in CT plans compared with TMR10-MRI. The average D100 and D95 for synthetic CT showed a 0.11% increase and 0.19% decrease compared with a 0.67% increase and 0.71% increase in CT-based compared to MRI plans. Overall, synthetic CT plans provided better coverage and a lower maximum dose.

**Table 1:** Dosimetric comparison of synthetic CT- and CT-based plans using convolution and regular clinical MRI with the TMR10 algorithm Lt = Left; Rt = Right; D100 = Dose covering 100% of target volume; D95 = Dose covering 95% of tumor volume; Min = Minimum; Max = Maximum; TMR = Tissue Maximum Ratio; MRI = Magnetic Resonance Imaging

Patient 1	TMR10				
	Max dose (Gy)	Min dose (Gy)	Mean dose (Gy)	D100 (Gy)	D95 (Gy)
Lt Temporal	30.88	15.46	21.46	15.43	17.1
Rt Frontal	30.9	14.61	24.25	14.61	18.7
Brain stem	5	0.56	1.41		
	CT Convolution				
	Max dose (Gy)	Min dose (Gy)	Mean dose (Gy)	D100 (Gy)	D95 (Gy)
Lt Temporal	29.96	13.13	20.25	13.1	16.6
Rt Frontal	30.83	30.83	19.08	19.03	20.6
Brain stem	4.3	0.57	1.42		
	Synthetic CT Convolution				
	Max dose (Gy)	Min dose (Gy)	Mean dose (Gy)	D100 (Gy)	D95 (Gy)
Lt Temporal	30.53	11.77	21.74	11.73	17.3
Rt Frontal	30.71	18.19	24.9	18.17	20.4
Brain stem	5.37	0.55	1.42		
Patient 2	MRI TMR10				
	Max dose (Gy)	Min dose (Gy)	Mean dose (Gy)	D100 (Gy)	D95 (Gy)
Lt Temporal	21.2	16.1	19.2	16.7	17.8
Rt Frontal 1	22.5	17.2	21.2	18	19.9
Rt Partial 1	26.6	17.5	24	18	21.3
Rt Partial 2	21.3	18.8	20.5	18	19.7
Rt Frontal 2	21.2	18.1	20.5	18.9	19.6
Rt Frontal 3	22.4	18.5	21.6	18	20.4
Lt Frontal 1	21.9	18.8	21	18	20.3
Rt Post-Frontal	35	14.9	23	15.4	17.8
Lt Frontal 2	21.2	18.1	20.5	18	19.6
	CT Convolution				
	Max dose (Gy)	Min dose (Gy)	Mean dose (Gy)	D100 (Gy)	D95 (Gy)
Lt Temporal	21.2	16.1	19	16.4	17.7
Rt Frontal 1	22.5	17.5	21.3	18	20.1
Rt Partial 1	26.4	17.8	24.1	18	21.3
Rt Partial 2	21.1	19	20.5	18	19.8
Rt Frontal 2	21.1	18.8	20.4	18	19.7
Rt Frontal 3	22.5	19	21.7	18	20.6
Lt Frontal 1	21.9	18.9	21.1	18	20.4
Rt Post-Frontal	34.7	14.4	22.8	15.5	17.6
Lt Frontal 2	21.1	18.4	20.5	18	19.7
	Synthetic CT Convolution				
	Max dose (Gy)	Min dose (Gy)	Mean dose (Gy)	D100 (Gy)	D95 (Gy)
Lt Temporal	21.2	16.2	19.1	16.7	17.7
Rt Frontal 1	22.5	17.4	21.3	18	20.1
Rt Partial 1	26.4	17.9	24.1	18	21.4
Rt Partial 2	21.1	18.9	20.5	18	19.8
Rt Frontal 2	21.1	18.9	20.5	18	19.8
Rt Frontal 3	22.5	19	21.8	18	20.6
Lt Frontal 1	21.9	18.9	21.1	18	20.4
Rt Post-Frontal	34.7	14.2	22.8	15.4	17.5
Lt Frontal 2	21.1	18.4	20.5	18	19.7
Patient 3	MRI TMR10				
	Max dose (Gy)	Min dose (Gy)	Mean dose (Gy)	D100 (Gy)	D95 (Gy)
Meningioma Rt	62.3	24	42.2	26.5	32.3
	CT Convolution				
	Max dose (Gy)	Min dose (Gy)	Mean dose (Gy)	D100 (Gy)	D95 (Gy)
Meningioma Rt	42.8	14.1	27.8	17.5	21.9
	Synthetic CT Convolution				
	Max dose (Gy)	Min dose (Gy)	Mean dose (Gy)	D100 (Gy)	D95 (Gy)
Meningioma Rt	62.5	24	42	26.2	32.1

## Discussion

For MRI-only radiotherapy, especially SRS planning, the use of synthetic CT images could potentially involve the application of automatic segmentation, the use of functional MR images, and even of computational texture analysis techniques such as radiomics. Very soon, larger MRI vendors, e.g. GE and Siemens, will offer FDA-approved MRI-only treatment planning platforms [24]. Generation of synthetic CT images and patient-specific distortion correction will be more accessible and streamlined, compatible with busy clinical workflow.

Thus, it is necessary to validate the accuracy of synthetic CT images and to ensure these images’ geometric and dosimetric accuracy is comparable with that of current CT images. Of necessity, this will involve the use of these images for RTP at different sites and both qualitative and quantitative evaluation of the accuracy of dose distribution. This procedure will be more accessible as more MRI-Linac machines become available. In this project, we looked at the application of synthetic CT images during SRS planning, a procedure in which MRI images are frequently used as a primary planning dataset.

Current SRS planning procedures involve the use of MR images without heterogeneity correction. Dose calculation on standard CT scans requires separate registration to MR for delineation of the target and normal tissue [1]. Synthetic CT images generated directly from MR have the potential to take advantage of the improved delineation clarity of MR while maintaining heterogeneity-corrected dose calculation within the treatment planning system. The use of synthetic CT has immediate applications for SRS planning, as well as future implications for developing real-time MR image guidance and plan adaptation for external beam radiotherapy. The incorporation of a convolution algorithm in newer SRS planning systems will improve dose calculation even in the face of tissue inhomogeneity, and especially in regions with air-tissue interfaces and sharp tissue changes.

## Conclusions

The results of this study show similarity between CT and synthetic CT-based SRS plans. Synthetic CT offered a noticeable improvement in target dose coverage and a more gradual dose fall off. The disadvantage is an increased dose (by 0.37%) to brainstem, an OAR, relative to plans created using clinical MRI images, which could be significant at higher doses. Future directions for this work will involve 1) further modification of the synthetic CT algorithm to improve its performance; 2) generation of a custom electron density curve to integrate synthetic CT images into the treatment planning system; and 3) study of the use of synthetic CT as the primary calculation image set for different types of treatment plan.
